# Coordinating the research response to COVID-19: Mali’s approach

**DOI:** 10.1186/s12961-020-00623-8

**Published:** 2020-09-17

**Authors:** Seydou Doumbia, Ydrissa Sow, Mahamadou Diakite, Chuen-Yen Lau

**Affiliations:** 1 University Clinical Research Center & Faculty of Medicine and Odontostomatology University of Sciences, Techniques and Technologies of Bamako, Bamako, Mali; 2 Collaborative Clinical Research Branch, Division of Clinical Research, National Institute of Allergy and Infectious Disease National Institutes of Health, 5601 Fishers Lane, Rockville, MD 20852 USA; 3 University Clinical Research Center & Faculty of Pharmacy, University of Sciences, Techniques and Technologies of Bamako, Bamako, Mali

**Keywords:** Mali, COVID-19, Research response, Public health, Coordination

## Abstract

Mali, like the rest of the world, has seen a rapid spread of COVID-19 since the first report of imported cases. Despite being a low-income country, Mali has leveraged scientific research resources via coordinated approaches to enable public health emergency planning and response to the COVID-19 pandemic. Mali’s approach includes the harmonization of research activities; leveraging of research laboratory capacity of the University Clinical Research Center, Mali International Center for Excellence and three other in-country laboratories for community COVID-19 testing; strengthening relationships amongst local and international stakeholders; and collaboration with the Ministry of Health to integrate scientific evidence into public policy and emergency management of COVID-19 through a platform of consultation and open communication. The country has implemented national coordination of its COVID-19 response by establishing a COVID-19 Scientific Advisory Committee and a COVID-19 Technical Coordination Committee, both within the Ministry of Health and working collaboratively with other stakeholders. Members of Mali’s COVID-19 Scientific Advisory Committee also serve as leaders of its principal academic and government clinical and public health research entities. This centralised approach has enabled the prioritisation of COVID-19 control activities, informed allocation of resources, evidence-based public health practices and timely decision-making in the pandemic setting. Though challenges remain, lessons learned from Mali’s harnessing of clinical research capacity to guide and support its COVID-19 response can be applied to future global health research challenges and illustrate the power of building public health-responsive research capacity in resource-limited settings through international collaboration.

## Introduction

Coronavirus disease 2019 (COVID-19) was first recognized in Wuhan, China, in December 2019 [1], though it was probably circulating earlier [2]. SARS-CoV-2, the etiologic agent of COVID-19, rapidly spread to diverse regions to cause a global pandemic with over 27 million cases and 890,000 deaths [3]. Short- and long-term impacts on the economy, infrastructure and social dynamics are vast. Currently, there is no known effective pre-exposure prophylaxis and treatment options are limited. The COVID-19 pandemic has created unprecedented demand for research to guide an evidence-based public health response. Thus, basic science as well as preclinical and clinical research towards these ends have accelerated quickly. Virologic and pathogenesis studies are informing the design of targeted interventions [4]. Animal model development will provide a platform for pre-clinical evaluation of potential interventions [5]. Human studies will clarify the epidemiology, disease course and efficacy of interventions. Longitudinal cohort studies are needed to fully understand the implications of COVID-19. Against this backdrop, it is important to align research priorities with local context, coordinate research activities by information sharing and ensure that activities consider currently available data.

Intimate research infrastructures in some developed countries may serve as models for the coordination of diverse and rapid research responses. For example, the Accelerating COVID-19 Therapeutic Interventions and Vaccines (ACTIV) Partnership, with members from government, academia and industry, will establish a collaborative framework for prioritising vaccine and therapeutic candidates in order to streamline clinical trials, tap into existing clinical trial networks, coordinate regulatory processes and leverage assets [6]. To mobilise the engineering community, the National Academies of Sciences, Engineering and Medicine has announced a call for innovative engineering solutions to COVID-19. These special COVID-19 task forces provide independent, objective analysis and guide public policy. While the current focus is COVID-19, concepts pertaining to the coordination of research activities and the use of research to inform policy apply to diverse infectious and non-infectious disease research topics. We herein share the approach to COVID-19 in Mali to demonstrate how challenges posed by the continuously evolving research landscape might be evaluated and managed.

## COVID-19 research is burgeoning

The breadth, intensity and volume of COVID-19 studies are dizzying. Maintaining awareness of current findings, integrating them into one’s research and avoiding the duplication of others’ efforts are continuous challenges. To this end, attempts to coordinate COVID-19 research around the world are in progress. Efforts in specific areas [7, 8], broad government efforts such as the ACTIV Partnership [9] and activation of the WHO R&D Blueprint to improve the global response support a comprehensive approach. As part of this strategy, we expect to see increasing linkages between public health and research activities. For instance, public health entities may screen for SARS-CoV-2 as part of their mitigation strategy. Cases may be referred for enrollment in clinical trials. Additionally, research serosurveys may be used to infer prior exposure patterns and inform the targeting of interventions. Mali is presently implementing observational studies and anticipates using these studies as a platform for intervention trials in the near future.

The conduct of protocols through networks and multi-centre international collaborations enhances study power, coordination and awareness. For example, WHO is conducting the Solidarity study in over 100 countries [10]. WHO provides access to study agents through public–private partnerships and works with developers and companies to ensure affordable access to efficacious products. However, large collaborations engender the challenges of harmonising activities across diverse settings. To this end, there may be heavy reliance on local partners who are familiar with their system’s nuances.

## Research environment in Mali

Mali is a low-income country in West Africa with an average life expectancy of approximately 59 years [11] and Malaria as a leading cause of mortality. Collaboration between the Mali and United States governments began in the late eighties and led to the creation of the Malaria Research and Training Center (MRTC) in 1992. The MRTC has since played a major role in malaria vector control, drug resistance and immunoepidemiologic research. MRTC’s accomplishments paved the way for the development of a large multidisciplinary research programme known as the Center for Research and Training on HIV/tuberculosis (TB) (SEREFO) in 2003. SEREFO has conducted sustainable laboratory and clinical research on bacterial and viral diseases beyond its initial focus on HIV and TB. In 2015, the University Clinical Research Center (UCRC) was created in Bamako, Mali, through an initiative between the United States National Institutes of Health (NIH) and the Malian Ministry of Health (MoH) and Ministry of Higher Education and University of Science, Techniques and Technology of Bamako (USTTB). With its immunology, molecular biology and Biosafety Level-3 laboratories, which are rare in the region, SEREFO has become an integral part of UCRC. The UCRC and MRCT together form the International Center for Excellence in Research in Mali (ICER Mali), which is an arm of the USTTB for biomedical and clinical research. UCRC serves as a core clinical research facility in Mali, providing human capital and infrastructure to support infectious disease research of local relevance.

The governance structure of UCRC includes a Governing Council, the Executive Committee and the Directorate. The Governing Council is composed of four members (the Rector of USTTB, representatives of the MoH and the Ministry of Higher Education and Research (MHER), and the Director of the United States NIH National Institute of Allergy and Infectious Diseases (NIAID) Division of Clinical Research) who provide strategic guidance. The Executive Committee oversees the research activities of UCRC and is composed of 10 members, including senior researchers representing USTTB, the MoH, the MHER and NIAID. UCRC’s mission is to develop sustainable capacity for the conduct of clinical research, nurture the next generation of clinical research scientists, disseminate research results and promote the utilisation of findings. UCRC receives funding from NIAID, the Government of Mali and through competitive research grants funded by diverse agencies. UCRC has over 150 staff members, including a dozen junior scientists trained in United States, European and African universities, and more than 20 MDs, PharmDs and PhDs. Through research and training grants, the UCRC collaborates with universities in the United States, Europe, Africa and Asia. Partners include Johns Hopkins University, George Washington University, Tulane University, Harvard and the University of Florida in the United States; the London School of Hygeine and Tropical Medicine, Oxford, the University of Bordeaux and the University of Copenhagen in Europe; and regional research institutions such as the University of Cape Town in South Africa, the University of Conakry in Guinea and the University of Ouagadougou in Burkina Faso. UCRC is also a member of several research consortiums, including the West African Clinical Research Consortium that includes Mali, Guinea, Liberia, Ivory Coast and Sierra Leone, aimed at advancing evidence-based regional epidemic preparedness and public policy.

UCRC adheres to international standards and is continuously enhancing capacity. UCRC collaborators have access to core resources but provide support for additional activities. Through this paradigm, UCRC offers value to collaborators by providing laboratory diagnostic capacity for emerging pathogens such as Ebola, dengue, yellow fever, and COVID-19, clinical trial operations, regulatory monitoring, data management and administrative assistance to support the protocol lifecycle. In return, collaborations further increase UCRC’s impact by expanding the research portfolio, building additional capacity and enhancing the integration with the global research community. Several international collaborations have been built on this groundwork with support from NIH, WHO, Gates Foundation and others. For example, the UCRC lab has played a key role in implementing diagnostics and evaluation of vaccines for Ebola as part of the Partnership for Research on Ebola Vaccinations [12, 13].

The core facilities of ICER Mali consist of key laboratories, a management centre and several field sites. The laboratories include an American College of Physicians certified lab as well as medical entomology, Biosafety Level-3 and geographic information systems facilities. A repository enables storage of research specimens. Internet connectivity is available throughout. The Mali Service Center, established in 2014, provides administrative and financial management. Field sites are located in Dongèuèbougou, Bancoumana and Oulessebougou, villages approximately 40, 70 and 300 km, respectively, outside of Bamako. In Bamako, Point G, Sokonafin and Koulouba serve as Ebola vaccine trial sites. Collaborators may support additional sites for their specific projects. Through the network of core and augmented sites, cohorts for studies on HIV, malaria, TB and Ebola are accessible.

ICER Mali collaborates with the MoH by providing diagnostic support for emerging infectious diseases, TB and HIV; performing surveillance of HIV and TB drug resistance; and monitoring insecticide resistance by major disease vectors. Building on ICER Mali’s history of collaboration with the MoH to guide national disease control programmes, UCRC is now playing a key role in the government response to COVID-19. The chair of the Scientific Advisory Committee (SAC), created by the MoH to strategically coordinate the scientific management of the COVID-19 pandemic, is from UCRC leadership. The scientific advisor to the COVID-19 Technical Coordination Committee (TCC), created by the Prime minister’s office to oversee implementation of the government response to the crisis, is also from UCRC leadership. In addition, UCRC leadership chairs the biomedical study section of the COVID-19 grant review committee appointed by the MHER to assist the National Center for Scientific and Technological Research (CNRST) in setting COVID-19 research priorities and distributing small grants to local research teams.

## COVID-19 in Mali

In anticipation of COVID-19 reaching Mali, UCRC had collaborated with the United States NIH to train staff scientists on COVID-19 diagnostics and procured 2000 test kits in January 2020. This preparation was instrumental in Mali’s response, enabling the timely and accurate identification of cases. Respiratory specimens from all suspected cases were tested at UCRC during the first month of the epidemic. Mali’s first two COVID-19 cases were diagnosed on 25 March 2020 despite Mali having closed all borders with the exception of permitting a few commercial flights bringing Malians home from Europe, 1 week earlier. The laboratory diagnostic capacity was subsequently expanded to the Laboratory of the National Institute of Public Health, *Centre d’infectiologie Charles Mérieux* (CICM) and *Laboratoire de Biologie Moléculaire Appliqué* after additional supplies were also donated by the French Institute for Research and Development, the German government, the West African Health Organization and Jack Ma. Treatment facilities were established to handle positive cases in three major Bamako hospitals: Dermatologic Hospital (20 beds), Point G hospital (100 beds) and Hospital du Mali (100 beds). Nearly 80% of identified cases have been concentrated in and around the capital city of Bamako, though disease has been detected throughout the country.

The spread of COVID-19 within Mali has stimulated research efforts toward the development of locally relevant management strategies. Though the IRB of USTTB typically reviews only 2–3 projects per month, they received 16 COVID-19 related proposals in 2 months (Table 1). In order to prioritise research activities while harnessing local capacity and simultaneously enriching global knowledge, the CNRST organised a meeting using the WHO R&D Blueprint strategy. This approach seeks to coordinate and accelerate research that can contribute to containing the epidemic and facilitate optimal care. The CNRST meetings involved local scientists of key research institutions in Mali, including ICER Mali/UCRC, the Department of Traditional Medicine, the CICM, the Malian Association of Traditional Therapists and Herbalists, the Malian Association for the Promotion of Research, Invention and Technological innovation, the NGO Pivot Santé Population and the Malian Academy of Sciences. Priority research areas identified include COVID-19 epidemiology, clinical characteristics, therapeutics including local natural products, risk factors for poor outcomes, community engagement, and strategies to optimise compliance with prevention and control interventions.

**Table 1:** COVID-19 research proposals reviewed by the Ethics Committee, April–May 2020

N°	Protocol Title
1	Multicenter study of nosocomial transmission of the SARS-CoV-2 virus (NOSO-COR project)
2	Personal and family adaptation to COVID-19 in Sub-Sahara African countries
3	Sero-epidemiological study on COVID-19 by age group of the population in Mali
4	SOLIDARITY STUDY, an international randomized trial of additional treatments for COVID-19 in hospital patients receiving local standard of care
5	Public health surveillance under COVID-19 in Mali
6	Research plan for surveillance and prevention of COVID-19 infection in rural areas in Mali
7	Clinical and virologic characterization of COVID-19 in Mali
8	COVID-19 pandemic: seroprevalence study of health workers in Mali
9	Evaluation of therapeutic and prevention approaches using chloroquine and hydroxychloroquine with or without azithromycin against COVID-19 infection in Mali
10	Ex vivo phenotypic screening of drugs on fresh SARS-CoV-2 isolates in Mali (COVIDEX VIVO)
11	COVID-19 Community Seroprevalence Study in Mali
12	International citizens project to assess compliance with public health measures and their impact on the COVID-19 epidemic
13	Psychosocial status of the staff of institutions for care of people living with HIV (PLHIV): action to reduce the risks of contamination by COVID-19
14	Implementation of public health measures during the COVID-19 pandemic in French-speaking Africa: the case of Mali
15	A phase II study to assess the safety, tolerance and efficacy of APIVIRINE (a medicinal plant product) in adults with COVID-19 in Mali (API-COVID-19)
16	Implementation of public health measures for vulnerable populations during the COVID-19 pandemic in French-speaking African countries in conflict

## Mali’s approach to managing COVID-19 activities

The spread of COVID-19 in Mali has stimulated both public health and research responses. These have occurred in tandem, with research being designed to address and support public health needs, and public health programmes and populations contributing to research. The harmonisation of activities is facilitated by key leaders having roles in both domains. To this end, Mali created the COVID-19 TCC under the leadership of the prime Minister’s office and the SAC to the MoH to provide strategic orientation and evidence-based guidelines for the management of the COVID-19 pandemic. The TCC oversees and coordinates general multisectoral response activities for the country (e.g. police, border security, veterinary services, national communications, academia and civil society). It is co-directed by the Coordinator of National COVID-19 Response and the Coordinator of the National Platform for Disaster Risk Management led by the Ministry of Homeland Security and Civilian Protection. TCC recommendations are reviewed and adopted during inter-ministerial meetings led by the Prime Minister. The SAC, chaired by UCRC leadership, is composed of scientists and clinical researchers who meet weekly to assess the status of the epidemic and make recommendations to the MoH on COVID-19 operational issues, including local standards of care and research priorities. SAC representatives participate at the Higher Council of National Defense to advise the President of Mali on strategic decisions regarding COVID-19, including curfew, school closures and lockdowns. Within the MoH, a complementary committee of subject matter experts from different MoH departments was created to oversee control and surveillance.

To finance rapid implementation of control measures, the government has created a National Fund to fight COVID-19. This fund is supported by the Malian government in conjunction with private sector donations. Its current balance is over US $8,563,300 and intended to cover the purchase of drugs, healthcare workers, personal protective equipment, hospital supplies and containment centres. In addition, CRNST was able to secure 229,000€ from The National Competitive Fund for Research and Technological Innovation, which is generated through tax revenue of 0.20% of collections. CNRST then set up a request for COVID-19 proposals, which received 41 applications within 2 weeks. Proposals included clinical studies on diagnosis, treatment and mental health; social and behavioural studies on socio-economic impacts and knowledge, attitudes and practices; evaluation of a new phytotherapeutic intervention; and innovations related to e-health surveillance.

The public health response has been fairly structured, as exemplified by the rapid establishment of three treatment centres in Bamako. However, the research response has been less organised. In the setting of overtaxed capacity, social distancing and desire of multiple partners to rapidly implement studies addressing diverse issues, the limited research infrastructure is challenged to effectively engage and coordinate with its enthusiastic partners and separate research groups with interests in Mali. Proposed COVID-19 projects with UCRC address general epidemiology, potential natural and pharmaceutical interventions, prevention strategies, and the impact on healthcare personnel. Some studies are specific to Mali, while others are conducted through international networks. There is also a general emerging infection study that was being finalised and implemented when COVID-19 emerged. This study serves to collect observational data on COVID-19 patients while more specific protocols are implemented.

Malian research teams internal and external to ICER Mali/UCRC continue to be solicited by international partners for collaboration on additional studies against the backdrop of efforts to effectively support the current portfolio. Proposals often overlap with each other and with existing projects in terms of scientific goals, populations of interest and required resources. Without clear coordination enabling the prioritisation of initiatives and strategic selection of study sites, consequences extend beyond inefficiency to potential ‘community fatigue’ when too many studies are conducted. In such cases, the study population may come to resent or disengage from the research [14]. Given that ICER Mali/UCRC sites have long-term local footprints associated with several longitudinal studies, the multitude of COVID-19 studies should be integrated into a unified picture that the community can embrace. The SAC will ameliorate some of the remaining harmonisation challenges, including the coordination of study sites, study populations, resources, information sharing and staffing for protocols. It will also accelerate Institutional Review Board and MoH regulatory processes.

## Steps toward integration

Mali’s COVID-19 research activities highlight the need for coordination, provide an example of how that might be approached and demonstrate the challenges associated with rapid expansion. The identification of COVID-19 committees involving researchers, medical providers, public health policy-makers and other key stakeholders and building on existing relationships to address current research gaps associated with COVID-19 have been key to helping investigators understand the existing needs and those already being addressed. To accomplish the unification of the research portfolio, the mission of a scientific committee can be extended beyond an advisory role. This group may serve as a centralised body for review and endorsement of all COVID-19 research initiatives before submission to the IRB. The committee may track the conduct of COVID-19 protocols, coordinate data sharing and integrate findings into public health programmes [15]. Researchers desirous of working in Mali will be required to obtain approval from the central committee and provide updates at regular intervals.

Despite progress toward a more efficient and relevant research portfolio, challenges such as communication gaps and resource deficiencies have become obvious as people and infrastructure are taxed beyond capacity. These are exacerbated by quarantine requirements. Our experience in Mali highlights several factors critical for coordination under such circumstances. Communication is of paramount importance. It has not always been smooth, largely due to key players having competing priorities. Existing relationships nurtured through years of collaboration have facilitated communication during this busy time. Team members in Mali and the United States are accessible to each other for urgent and routine issues; standing calls amongst project leaders provide a forum for updates, questions and concerns to be incorporated into the paradigm. Trust and transparency are additional attributes that sustain teams during stress, particularly while finding novel work approaches that maintain social distance. Partners with experience relying on each other are able to build on prior interactions to focus on the science [16]. They must employ flexible approaches, such as developing expedited protocol approval pathways and remote implementation strategies, to accommodate new ideas and challenges.

Having an established core in Mali and shared goals amongst key partners has made rapid expansion of Mali’s COVID-19 research portfolio possible. Notwithstanding growing pains, these provide a framework on which collaborations can be built and nurtured. The close link with the Malian MoH, including leadership that hold positions in both the MoH and in research entities, connects the framework to public health interests. Thus, studies of greatest relevance can be prioritised.

Case Study: Orchestration of COVID-19 research, public health and policy efforts The head of Mali’s COVID-19 Scientific Advisory Committee also serves as head of its primary academic and government clinical research entity, the UCRC Mali Collaboration described in the text. While this ensures network awareness of ongoing and proposed research in Mali, associated time commitments may strain other activities. Nonetheless, centralised knowledge of the research portfolio has enabled the identification of overlapping proposals and connecting of investigators who may collaborate synergistically. It also supports the informed allocation of limited resources. Even when investigators do not conduct a study together, they may still concatenate data through centralised relationships.

## Conclusions

COVID-19 research will further expand in Mali and globally. Some work will support hypothesis generation such as the characterisation of COVID-19 in the general population. Subsequently, findings in subpopulations, such as those with HIV co-infection, can be evaluated in follow-up studies. These cohorts may then engage in treatment and prevention trials to inform public health approaches. Mali will inevitably bring on new partners, which will enable the coordination of internal activities with new partners’ portfolios.

Lessons learned from COVID-19 research, including its influence on Mali’s response to the epidemic, can be applied to future global research challenges. ICER Mali/UCRC exemplifies collaborative efforts via its unique pathway for knowledge and technology exchange between resource-limited settings and the developed world. Paramount to deploying an effective and efficient research response is communication amongst stakeholders. Maintaining an operational clinical research core built through an international government-to-government collaboration between the United States and Mali, the availability of research infrastructure and the accessibility of local expertise developed with mentorship from international research partners have been instrumental to Mali’s rapid evidence-based response to COVID-19. Nonetheless, effective use of these assets requires coordination when multiple efforts involving multiple partners are being made toward the same end. Over the past few months, this need has been increasingly recognised in the global COVID-19 research community and central sources of information have been developed [17, 18]. Mali will work through its COVID-19 SAC and TCC, both within the MoH, to affect a harmonised national research response to COVID-19 in concert with the world’s response.

## Data Availability

Data sharing is not applicable to this article as no datasets were generated or analysed during the current study.

## Abbreviations

ACTIV: Accelerating COVID-19 Therapeutic Interventions and Vaccines
CICM: *Centre d’infectiologie Charles Mérieux*
CNRST: National Center for Scientific and Technological Research
COVID-19: Coronavirus disease 2019
ICER Mali: International Center for Excellency in Research in Mali
MHER: Ministry of Higher Education and Research
MoH: Ministry of Health
MRTC: Malaria Research and Training Center
NIAID: National Institute of Allergy and Infectious Diseases
NIH: National Institutes of Health
SAC: Scientific Advisory Committee
SEREFO: Center for Research and Training on HIV/TB
TB: Tuberculosis
TCC: Technical Coordination Committee
UCRC: University Clinical Research Center
USTTB: University of Science, Techniques and Technology of Bamako

## References

[1] Paules CI, Marston HD, Fauci AS. Coronavirus infections-more than just the common cold. JAMA. 2020. doi: 10.1001/jama.2020.0757. 10.1001/jama.2020.0757 31971553

[2] Li X, Zai J, Zhao Q (2020). Evolutionary history, potential intermediate animal host, and cross-species analyses of SARS-CoV-2. J Med Virol.

[3] Dong E, Du H, Gardner L (2020). An interactive web-based dashboard to track COVID-19 in real time. Lancet Infect Dis.

[4] Lan J, Ge J, Yu J (2020). Structure of the SARS-CoV-2 spike receptor-binding domain bound to the ACE2 receptor. Nature.

[5] Shi J, Wen Z, Zhong G (2020). Susceptibility of ferrets, cats, dogs, and other domesticated animals to SARS-coronavirus 2. Science.

[6] Collins FS, Stoffels P. Accelerating COVID-19 Therapeutic Interventions and Vaccines (ACTIV): an unprecedented partnership for unprecedented times. JAMA. 2020. 10.1001/jama.2020.8920. 10.1001/jama.2020.8920 32421150

[7] Holmes EA, O'Connor RC, Perry VH (2020). Multidisciplinary research priorities for the COVID-19 pandemic: a call for action for mental health science. Lancet Psychiatry.

[8] Tsou IYY, Liew CJY, Tan BP (2020). Planning and coordination of the radiological response to the coronavirus disease 2019 (COVID-19) pandemic: the Singapore experience. Clin Radiol.

[9] NIH to launch public-private partnership to speed COVID-19 vaccine and treatment options [press release]. https://www.nih.gov/news-events/news-releases/nih-launch-public-private-partnership-speed-covid-19-vaccine-treatment-options. 2020. Accessed 7 Sept 2020.

[10] Alpern JD, Gertner E (2020). Off-label therapies for COVID-19 – are we all in this together?. Clin Pharmacol Ther.

[11] World Bank. Life Expectancy at Birth. https://data.worldbank.org/indicator/SP.DYN.LE00.IN?locations=ML. Accessed 7 Sept 2020.

[12] Diarra B, Safronetz D, Sarro YD (2016). Laboratory response to 2014 Ebola virus outbreak in Mali. J Infect Dis.

[13] Levy Y, Lane C, Piot P (2018). Prevention of Ebola virus disease through vaccination: where we are in 2018. Lancet.

[14] Too many academics study the same people. Nature. 2017;551(7679):141–2. 10.1038/551141b. 10.1038/551141b 29120432

[15] Grue L, Siddiqui S, Limmathurotsakul D, Kamaludi A, Karyana M, Lau CY (2016). Commentary: data sharing in South East Asia. BMJ.

[16] Lau CY, Wang C, Orsega S (2014). International Collaborative Research Partnerships: blending science with management and diplomacy. J AIDS Clin Res.

[17] Kaiser J (2020). NIH organizes hunt for drugs. Science.

[18] Thorlund K, Dron L, Park J, Hsu G, Forrest JI, Mills EJ. A real-time dashboard of clinical trials for COVID-19. Lancet Digital Health. 2020;2(6):e286–e287. https://www.thelancet.com/journals/landig/article/PIIS2589-7500(20)30086-8/fulltext. 10.1016/S2589-7500(20)30086-8 PMC7195288 32363333

